# A Multi-Antigen Broad-Spectrum Coronavirus Vaccine Induces Potent and Durable Cross-Protection Against Infection and Disease Caused by Multiple SARS-CoV-2 Variants

**DOI:** 10.21203/rs.3.rs-7208748/v1

**Published:** 2025-08-20

**Authors:** Swayam Prakash, Nisha R. Dhanushkodi, Afshana Quadiri, Hawa Vahed, Aziz A. Chentoufi, Pierre-Gregoire Coulon, Izabela Coimbra Ibraim, Assia El Babsiri, Delia F. Tifrea, Cesar J. Figueroa, Daniel Gil, Jeffrey B. Ulmer, Lbachir BenMohamed

**Affiliations:** University of California Irvine; University of California Irvine; University of California Irvine; University of California Irvine; University of California Irvine; University of California Irvine; University of California Irvine; University of California Irvine; University of California Irvine; University of California Irvine; TechImmune, LLC, University Lab Partners; TechImmune, LLC, University Lab Partners; University of California Irvine

**Keywords:** pan-Coronavirus vaccine, SARS-CoV-2, COVID-19, Variants of concern, Cross-protective, CD4+ T cells, CD8+ T cells

## Abstract

The first generation of Spike-based COVID-19 vaccines has reduced the risk of hospitalization, serious illness, and death caused by SARS-CoV-2 infections. However, waning immunity induced by these vaccines has failed to prevent immune escape, resulting in the emergence of multiple variants of concern (VOCs) and the prolongation of the COVID-19 pandemic. We hypothesize that a next-generation Coronavirus (CoV) vaccine incorporating highly conserved SARS-CoV-2 T cell antigens would confer potent, broad, and long-lasting cross-protective immunity against multiple VOCs. In the present study, we identified ten non-Spike antigens that are common and highly conserved among 8.7 million SARS-CoV-2 strains, twenty-one VOCs, SARS-CoV-1, MERS-CoV, seasonal human common cold CoVs, and animal CoVs. Seven of the ten antigens were preferentially recognized by CD8^+^ and CD4^+^ T cells from unvaccinated asymptomatic COVID-19 patients, irrespective of VOC infection. Three out of the seven conserved T cell antigens (NSP2, NSP14, and Nucleocapsid), when administered to golden Syrian hamsters: (*i*) Induced high frequencies of lung-resident polyfunctional antigen-specific CXCR5^+^CD4^+^ T follicular helper (T_FH_) cells, GzmB^+^CD4^+^ and GzmB^+^CD8^+^ cytotoxic T cells (T_CYT_), and CD69^+^IFN-g^+^TNFa^+^CD4^+^ and CD8^+^ effector T cells (T_EFF_); (*ii*) Reduced morbidity, viral load, lung pathology, and COVID-19-like symptoms caused by various VOCs, including the highly pathogenic B.1.617.2 Delta variant and the recently circulating KP.3 Omicron variant; (*iii*) Improved protection conferred by spike-alone mRNA, and (iv) Conferred protection that last for more than one year post-vaccination. This multi-antigen CoV vaccine could be adapted for clinical use to confer potent, broad-spectrum, and durable cross-protective immunity against current and future variants of concern.

## INTRODUCTION

The Coronavirus disease 2019 (COVID-19) pandemic has created one of the most significant global health crises in nearly a century^1, 2, 3, 4, 5, 6^. The first-generation Spike-based COVID-19 vaccines have contributed to reducing the COVID-19 pandemic and mitigating symptoms and deaths from SARS-CoV-2 infection^7, 8^. However, since early 2020, breakthrough infections due to continued transmission have led to the emergence of VOCs and surges in hospitalizations, illnesses, and deaths, straining the world's healthcare systems^9, 10^. Today, thousands of patients are still being hospitalized and dying every month from COVID-19^11^. At the same time, ~ 10–30% of infected individuals with mild to moderate infections and up to 50% of people with severe infections carry the burden of long COVID, a debilitating chronic disease that currently affects over 10 million people in the U.S. and 410 million people globally, with no approved treatments^12, 13, 14, 15^. Hence, a superior next-generation vaccine is desperately needed.

In response to immune escape and waning immunity to Spike-based COVID-19 vaccines, these first-generation vaccines have been periodically updated to incorporate the Spike mutations of the new variants that continue to emerge^16^. This vaccine strategy of chasing the variants and sub-variants cannot keep pace with the fast-emerging and rapidly mutating Omicron lineages^16^, as the sequences of the Spike protein, such as in the recently circulating Omicron subvariants KP.3, NB.1.8.1, and LP.8.1 Omicron subvariants, have already undergone over 100 accumulated mutations, diverging from the XBB1.5-adapted bivalent vaccine^17, 18, 19^. As a consequence, the bivalent vaccine introduced in 2022 was only effective 4 to 29% against the Omicron subvariants, circulating that winter^17, 18, 19^, and its effectiveness decreased even further against the more recent divergent and highly transmissible EG.5, HV.1, JN.1, JN.3, KP.3, NB.1.8.1, and LP.8.1 Omicron subvariants, circulating in the winters of 2024–2025^17, 18, 19^. These observations underscore the need for a superior next-generation CoV vaccine strategy that induces broad, durable, cross-protective immunity^20, 21, 22^ and thereby halts the ongoing COVID-19 pandemic, as well as provides protection against future SARS-CoV-2 variants^23^.

Recently, our group and others have: (*i*) Identified specific sets of highly conserved SARS-CoV-2 non-Spike antigens targeted by frequent cross-reactive functional CD4^+^ and CD8^+^ T cells from asymptomatic COVID-19 patients (i.e., unvaccinated individuals who never develop any COVID-19 symptoms despite being infected)^3, 5, 24, 25, 26, 27, 28, 29, 30^; (*ii*) Discovered that increased frequencies of lung-resident CD4^+^ and CD8^+^ T cells specific to common CoV antigens protected against multiple SARS-CoV-2 VOCs in mouse models^1, 3, 31, 32^; (*iii*) Demonstrated that enriched cross-reactive lung-resident memory CD4^+^ and CD8^+^ T_RM_ cells that selectively target early-transcribed SARS-CoV-2 antigens from the replication and transcription complex (RTC) region are associated with a rapid clearance of infection in so-called “SARS-CoV-2 aborters” (i.e., unvaccinated SARS-CoV-2 exposed seronegative individuals who rapidly abort the virus replication)^33, 34, 35, 36, 37^. We hypothesize that a next-generation CoV vaccine that incorporates highly conserved and early-expressed RTC antigens selectively targeted by CD4^+^ and CD8^+^ T cells from asymptomatic COVID-19 patients and “SARS-CoV-2 aborters” would confer stronger, broader, and longer-lasting protective immunity against rapidly transmissible and highly pathogenic VOCs.

In the present study, using *in-silico* bioinformatic techniques, we identified non-Spike RTC antigens that are highly conserved in 8.7 million genome sequences of SARS-CoV-2 strains that have circulated worldwide, including twenty-one VOCs, SARS-CoV-1, MERS-CoV, seasonal common cold coronaviruses, and animal CoVs (i.e., Bats, Civet Cats, Pangolins, and Camels). Seven non-Spike highly conserved antigens were selectively recognized by cross-reactive CD4^+^ and CD8^+^ T cells from unvaccinated asymptomatic COVID-19 patients. Three of these seven T cell antigens, when delivered as mRNA/LNP, safely induced superior B and T_RM_ cell cross-protective immunity compared to spike-alone mRNA vaccines, which lasted more than 12 months post-vaccination against several pathogenic and heavily mutated SARS-CoV-2 variants and sub-variants in the hamster model. These findings provide critical insights into the development of B and T cell antigen-based CoV vaccines that can confer broad and long-lasting, cross-protective immunity against highly mutated and pathogenic VOCs.

## RESULTS

### 1. Five highly conserved regions that encode ten common structural, non-structural, and accessory protein antigens were identified in the SARS-CoV-2 genome.

**Figure 1:**
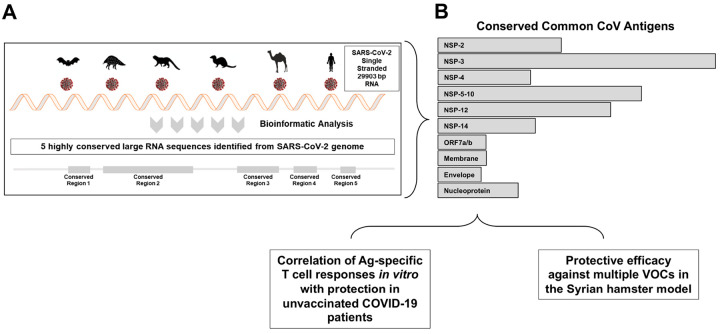
Highly conserved non-spike, structural, non-structural, and accessory protein antigens identified in the SARS-CoV-2 genome: (**A**) Bioinformatic analysis and alignment of the 29,903 bp single strand RNA of 8.7 million genome sequences of SARS-CoV-2 strains that circulated worldwide over the last 5 years, including 20 VOCs; SARS-CoV; MERS-CoV; common cold Coronaviruses; and twenty-five animal’s SARS-like Coronaviruses (SL-CoVs) genome sequences isolated from bats (Rhinolophus affinis, Rhinolophus malayanus), pangolins (Manis javanica), civet cats (Paguma larvata), and camels (Camelus dromedaries). Shown in light gray are five highly conserved regions identified from the SARS-CoV-2 genome sequences. (**B**) Depicts 10 highly conserved non-Spike antigens that comprise three structural (Membrane, Envelope, and Nucleoprotein), 12 non-structural (NSP-2, NSP-3, NSP-4, NSP-5–10, NSP-12, and NSP-14), and one accessory protein (ORF7a/b) as potential T cell antigens used to construct the individual and combined mRNA/LNP vaccines. The boxes illustrate the strategies used to evaluate the potential of these T cell antigens to confer protective immunity.

The SARS-CoV-2 single-stranded genome comprises 29,903 base pairs (bp), which encodes 29 proteins, including four structural, sixteen nonstructural, and nine accessory regulatory proteins^38^. Using several *in-silico* bioinformatic approaches and alignments of 8.7 million genome sequences of Coronavirus strains that circulated worldwide throughout the pandemic, including all the previously designated Variants of Concerns (VOC), Variants of Interests (VOI), Variants being Monitored (VBM); SARS-CoV-1; MERS-CoV; Common Cold Coronaviruses (i.e., α-CCC-229E, α-CCC-NL63, βCCC-HKU1, and βCCC-OC43 strains); and twenty-five animal SARS-like CoVs (SL-CoVs) genome sequences isolated from bats, pangolins, civet cats, and camels, we identified five highly conserved regions in the SARS-CoV-2 single-stranded RNA genome (1–1,580bp, 3,547 − 12,830bp, 1772–21,156bp, 22,585 − 24,682bp, and 26,660 − 27,421bp, Fig. 1). Further Sequence Homology Analysis confirmed that the five SARS-CoV-2 genome regions encode for ten highly conserved non-Spike T cell antigens (NSP-2 (Size: 1,914 bp, Nucleotide Range: 540 bp – 2,454 bp), NSP-3 (Size: 4,485 bp, Nucleotide Range: 3,804 bp – 8,289 bp), NSP-4 (Size: 1,500 bp, Nucleotide Range: 8,290 bp – 9,790 bp), NSP-5–10 (Size: 3,378 bp, Nucleotide Range: 9,791 bp – 13,169 bp), NSP-12 (Size: 2,796 bp, Nucleotide Range: 13,170 bp – 15,966 bp), NSP-14 (Size: 1,581 bp, Nucleotide Range: 17,766 bp – 19,347 bp), ORF7a/b (Size: 492 bp, Nucleotide Range: 27,327 bp – 27,819 bp), Membrane (Size: 666 bp, Nucleotide Range: 26,455 bp – 27,121 bp), Envelope (Size: 225 bp, Nucleotide Range: 26,177 bp – 26,402 bp), and Nucleoprotein (Size: 1,248 bp, Nucleotide Range: 28,206 bp – 29,454 bp) (Fig. 1). The gene sequences of these ten highly conserved antigens were then used to design and construct N1-methylpseudouridine (m1ψ)-modified mRNAs encapsulated in lipid nanoparticles (mRNA/LNP) for assessment of safety, immunogenicity, and protective efficacy against several SARS-CoV-2 VoCs in the golden Syrian hamster model (Fig. 1).

Mutations screened against twelve major SARS-CoV-2 variants and sequence homology analysis confirmed the sequences representing the ten non-Spike antigens are highly conserved in the currently highly mutated BA.2.86, JN.1, KP.3, NB.1.8.1, and LP.8.1 Omicron sub-variants. As expected, with 346 cumulative mutations since the ancestral Wuhan strain, the sequence of the Spike is heavily mutated in the latest Omicron sub-variants compared to the non-Spike antigens. The sequences of the Spike protein have new mutations in the current highly transmissible and most immune-evasive Omicron sub-variants. While the spike protein of Omicron BA.2.86 has a total of 61 non-synonymous mutations, OmicronJN.1 and Omicron KP.3 have 62 and 65 spike-specific non-synonymous mutations, respectively.NB.1.8.1LP.8.1. In contrast, compared to Spike, the NSP-2 has two common mutations (A211D and P314L), and Nucleocapsid has eight common mutations shared among Omicron BA.2.86, JN.1, and KP.3 variants (P13L, E31-, R32-, S33-, R203K, G204R, Q229K, S413). Whereas no non-synonymous mutation for NSP-14 has been reported for any of these Omicron sub-variants.NB.1.8.1LP.8.1 Notably, the sequence of NSP-14 is fully conserved (100%) in all variants and subvariants, including the recent JN.1, KP.3, NB.1.8.1, and LP.8.1, which supports the vital role of these antigens in the life cycle of SARS-CoV-2. Of the ten non-Spike antigens, Nucleoprotein was the least conserved in all variants and sub-variants, but remains an important vaccine target, as it is the most abundant viral protein and one of the most predominantly targeted antigens by T cells in individuals with less severe COVID-19 disease^39, 40^.

### 2. Human memory CD4^+^ and CD8^+^ T cells preferentially target seven of the ten highly conserved SARS-CoV-2 antigens and correlate with improved disease outcome in unvaccinated asymptomatic COVID-19 patients:

We next determined whether the ten highly conserved non-Spike antigens are targeted by CD4^+^ and CD8^+^ T cells from “naturally protected” unvaccinated COVID-19 patients.

**Figure 2:**
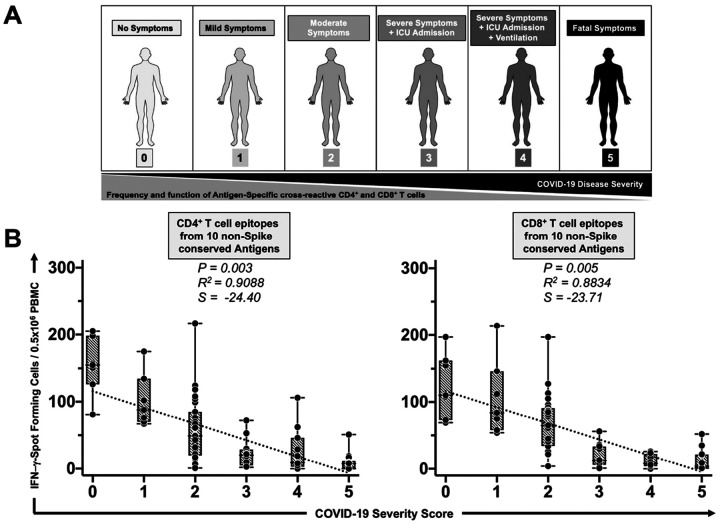
IFN-g-producing CD4^+^ and CD8^+^ T cell responses to highly conserved antigens in unvaccinated COVID-19 patients with various degrees of disease severity: (**A**) Illustrates the degrees of severity of COVID-19 disease in unvaccinated COVID-19 patients (*n* = 71), as divided into six groups scored 0 to 5 and described in Materials and Methods (Black = severity 5, to white = severity 0). (**B**) PBMCs from HLA-DRand HLA-A*0201-positive COVID-19 patients were isolated and stimulated for a total of 72 hours with a pool of peptides corresponding to CD4^+^ and CD8^+^ T cell epitopes from the ten selected conserved antigens (i.e., NSP-2, NSP-3, NSP-4, NSP-5–10, NSP-12, NSP-14, ORF7a/b, Membrane, Envelope, and Nucleoprotein). The number of IFN-g-producing T cells was quantified in each of the 71 patients using an ELISpot assay. Shown are the average/mean numbers (± SD) of IFN-g-spot forming cells (SFCs) for CD4^+^ (*left panel*) or CD8^+^ (*right panel*) T cell responses divided into six groups based on disease severity. PHA was used as a positive control of T-cell activation. Unstimulated negative control SFCs were subtracted from the SFC counts of peptide-stimulated cells. For all graphs, the coefficient of determination (R^2^) is calculated from the Pearson correlation coefficients. The associated *P*-value and the slope (S) of the best-fitted line (dotted line) are calculated using linear regression analysis and are indicated. The gray-hatched boxes in the correlation graphs extend from the 25^th^ to the 75th percentiles (hinges of the plots), with the median represented as a horizontal line in each box, and the extremities of the vertical bars showing the minimum and maximum values. Results are representative of two independent experiments and were considered statistically significant at *P* ≤ 0.05 using either the Mann-Whitney test (two groups) or the Kruskal-Wallis test (more than two groups).

CD4^+^ and CD8^+^ T cell responses specific to highly conserved epitopes, selected from these non-Spike antigens, were compared in unvaccinated asymptomatic individuals (those individuals who never developed any COVID-19 symptoms despite being infected with SARS-CoV-2) versus unvaccinated symptomatic COVID-19 patients (those patients who developed severe to fatal COVID-19 symptoms) (Fig. 2A). Unvaccinated HLA-DRB1*01:01^+^ and HLA-A*0201 COVID-19 patients (*n* = 71) enrolled between January 2020 and December 2023, irrespective of the variant of concern with which they were infected, were divided into six groups based on the level of severity of their COVID-19 symptoms (increasing severity from 0 to 5), assessed at discharge (Fig. 2A). The clinical and demographic characteristics of this cohort of COVID-19 patients are detailed in our previous publication^41^. Fresh PBMCs were isolated and then stimulated *in vitro* for 72 hours using recently identified highly conserved 13 HLA-DR-restricted CD4^+^ or 16 HLA-A*0201-restricted CD8^+^ T cell peptide epitopes derived from the non-structural proteins (NSPs), the ORF7a//b, Membrane, Envelop, and Nucleoprotein, as detailed in Materials & Methods. The number of IFN-γ-producing CD4^+^ T and CD8^+^ T cells specific to epitopes from all ten selected conserved antigens is shown in Fig. 2B. Specifically, thirteen individual cross-reactive CD4^+^ T cell epitopes (**Supplementary Fig. S2**) and sixteen individual cross-reactive CD8^+^ T cell epitopes (**Supplementary Fig. S3**) from the selected ten highly conserved antigens were quantified in each of the six groups of COVID-19 patients using an ELISpot assay (i.e., number of IFN-γ-spot forming T cells or “SFCs”). We then performed Pearson correlation analyses to determine the linear correlation between the magnitude of CD4^+^ and CD8^+^ T cell responses directed toward each of the conserved SARS-CoV-2 epitopes versus the severity of subsequent COVID-19 disease. A correlation is considered strong when the coefficient R-value is between 0.7 and 1.

Overall, the highest frequencies of epitope-specific IFN-g-producing CD4^+^ and CD8^+^ T cells (determined as mean SFCs > 50 per 0.5 × 10^6^ PBMCs fixed as threshold) were detected in the unvaccinated COVID-19 patients who developed less severe disease (i.e., severity 0, 1, and 2, Figs. 2B, **Supplementary Fig. S2**, and **Supplementary Fig. S3**). In contrast, the lowest frequencies of IFN-γ-producing CD4^+^ and CD8^+^ T cells were detected in unvaccinated COVID-19 patients who subsequently developed severe disease (severity scores 3 and 4, mean SFCs < 50) and death (severity score 5, mean SFCs < 25). We found a strong positive linear correlation between the high magnitude of IFN-γ-producing CD4^+^ and CD8^+^ T cells specific to seven out of ten common T cell antigens and the “natural protection” observed in unvaccinated asymptomatic COVID-19 patients (Fig. 2B, **Supplementary Fig. S2**, and **Supplementary Fig. S3**). This positive correlation existed irrespective of whether CD4^+^ and CD8^+^ T cells target structural, non-structural, or accessory regulatory SARS-CoV-2 antigens.

Taken together, these results: (*i*) Demonstrate an overall higher magnitude of CD4^+^ and CD8^+^ T cell responses specific to conserved non-Spike antigens present in unvaccinated asymptomatic COVID-19 patients irrespective of the SARS-CoV-2 variant of concern to which they were exposed; (*ii*) Suggest a crucial role of these seven highly conserved structural, non-structural, and accessory regulatory T cell antigens, in protection from symptomatic and fatal Infections caused by multiple variants; and (*iii*) Support these conserved non-Spike Coronavirus antigens as potential targets for a broad-spectrum CoV vaccine.

### 3. Conserved SARS-CoV-2 NSP-2-, NSP-14- and Nucleoprotein-based mRNA/LNP vaccines confer protection against the highly pathogenic Delta variant (B.1.617.2):

We constructed methylpseudouridine–modified (m1Ψ) mRNAs encoding each of the ten highly conserved T cell antigens (i.e., NSP-2, NSP-3, NSP-4, NSP-5–10, NSP-12, NSP-14, ORF7a/b, Membrane, Envelope, and Nucleoprotein), based on the Omicron sub-variant BA.2.75, using the CleanCap technology^42^. mRNA vaccines expressing the prefusion Spike protein stabilized by either two (Spike 2P) or six (Spike 6P) prolines were constructed as positive controls for B cell immunity^43, 44^. The twelve mRNA vaccines were then encapsulated in lipid nanoparticles (LNPs)^45^. The mRNA/LNP platform was selected as the antigen delivery technology, as clinically proven Spike mRNA/LNP-based vaccines have been validated for safety and efficacy in humans and can be manufactured on a large scale to support rapid and global mass vaccination.

**Figure 3:**
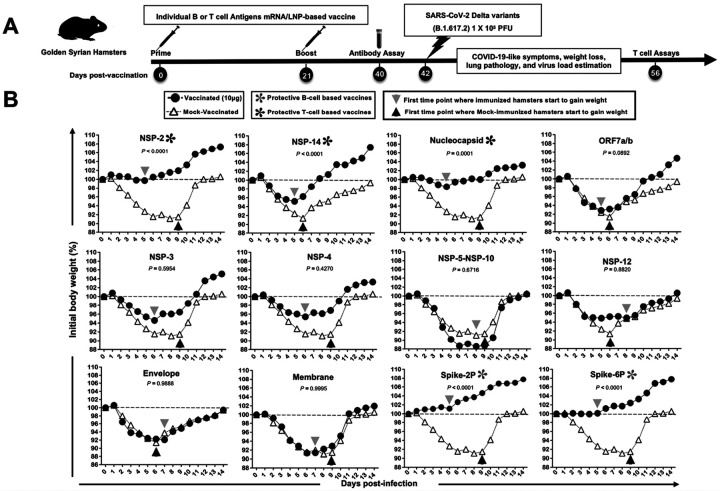
Screening of 10 highly conserved T cell antigens for protection against the highly pathogenic Delta variant (B.1.617.2) in golden Syrian hamsters: (**A**) Experimental plan to screen for vaccine efficacy. Male hamsters (*n* = 6 per group) were immunized intramuscularly on day 0 (prime) and day 21 (boost) with 10 mg/dose of the mRNA/LNP-based Coronavirus vaccines individually expressing the 10 highly conserved non-Spike T-cell antigens. Hamsters that received phosphate-buffered saline alone were used as mock-immunized controls (*Saline*, *Mock*, *n* = 6). Three weeks after booster vaccination (day 42), vaccinated and mock-vaccinated hamsters were intranasally challenged (both nostrils) with 1 × 10^5^ pfu of SARS-CoV-2 highly pathogenic Delta variant (B.1.617.2). Weight losses were assessed for 14 days post-challenge. (**B**) Shows percent weight change for 14 days post-challenge normalized to the initial body weight on the day of infection in hamsters immunized with mRNA/LNP expressing NSP-2, NSP-3, NSP-4, NSP-5–10, NSP-12, NSP-14, ORF7a/b, Membrane, Envelope, Nucleoprotein, Spike 2P, and Spike 6P at 10 mg/dose. The dashed line indicates the 100% starting body weight. The arrowheads indicate the first day post-challenge when the weight loss is reversed in T cell- and Spike antigen-vaccinated (*grey arrowhead*) and mock-vaccinated (*black arrowhead*) hamsters. The data represent two independent experiments; the graphed values and bars represent the standard deviation (SD) between the two experiments. Mann-Whitney test (for two groups) or the Kruskal-Wallis test (for more than two groups) was used for statistical analysis.

To screen for protective efficacy, each of the mRNA/LNP-based vaccines were delivered individually by the intramuscular route to outbred golden Syrian hamsters, then challenged with the highly pathogenic Delta variant (B.1.617.2) (Fig. 3A). Golden Syrian hamsters are naturally susceptible to SARS-CoV-2 infection, owing to the high degree of similarity between hamster ACE2 and human ACE2 (hACE2), and develop symptoms of COVID-19-like disease that closely mimic the COVID-19 pathogenesis in humans^46, 47, 48, 49, 50^. Male golden Syrian hamsters (*n* = 6 per group) were immunized intramuscularly on day 0 (prime) and day 21 (boost) with individual mRNA/LNP-based vaccines at a 10 μg/dose (Fig. 3A), based on previous similar mRNA-LNP vaccine studies in mice and hamsters^40, 51^. Hamsters that received phosphate-buffered saline alone were used as mock-immunized controls (*Saline*, *Mock*, *n* = 6). Power analysis demonstrated that five hamsters per group were sufficient to produce significant results with a power > 80%. Three weeks after the second immunization, hamsters were challenged intranasally with the SARS-CoV-2 Delta variant (B.1.617.2) (1 × 10^5^ pfu total in both nostrils). The dose of challenge virus was determined based on titration studies comparing 5 × 10^4 pfu, 1 × 10^5 pfu, and 5 × 10^5 pfu. pfu, with the middle dose of 1 × 10 ^5 pfu being sufficient to produce substantial disease in hamsters (data *not shown*).

**Figure 4:**
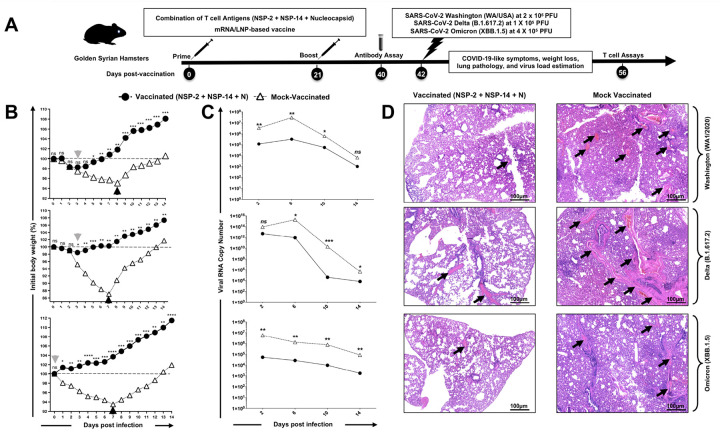
Protection against multiple SARS-CoV-2 variants induced by combined NSP-2, NSP-14, and Nucleoprotein-based mRNA/LNP vaccine in the hamster model: (**A**) Experimental design and timeline to study the vaccine efficacy in golden Syrian hamsters. Male hamsters were immunized intramuscularly twice on day 0 (prime) and day 21 (boost) with the combined NSP-2, NSP-14, and Nucleoprotein-based mRNA/LNP vaccine (*n* = 6 per group) or mock-vaccinated (*Mock*, *n* = 6 per group). Three weeks after booster vaccination (day 42), vaccinated and mock-vaccinated hamsters were intranasally challenged (both nostrils) with, 2 × 10^5^ pfu of the wild-type Washington variant (WA1/2020), 1 × 10^5^ pfu of the highly pathogenic Delta variant (B.1.617.2) or 2 × 10^5^ pfu of the highly transmissible Omicron sub-variant (XBB1.5). COVID-19 disease parameters, including (**B**) initial body percent weight loss, (**C**) viral RNA copy number, and (**D**) lung pathology, were assessed for 14 days post-challenge. (**B**)_Shows percent weight change for 14 days post-challenge, normalized to the initial body weight on the day of infection for each variant and sub-variant. The dashed line indicates the 100% starting body weight. The arrowheads indicate the first day post-challenge when the weight loss is reversed in T cell- and Spike antigen (*grey arrowhead*) vaccinated and mock (*black arrowhead*) vaccinated hamsters. (**C**) Two-, 6-, 10-, 14-days post-infection (p.i.) with the wild-type Washington variant (WA1/2020), the highly pathogenic Delta variant (B.1.617.2), or the highly transmissible Omicron sub-variant (XBB1.5), viral loads were analyzed, to evaluate vaccine-induced protection against virus replication, by comparing viral RNA copies in the hamster's throats and lungs between mock and vaccine groups. Viral RNA copies were quantified by RT-PCR and expressed as log_10_ copies per milligram of throat tissue. The graphs show a comparison of viral titers in the lungs of vaccinated and mock-vaccinated hamsters. (**D**) Representative H & E staining images of lung pathology at day 14 p.i. of SARS-CoV-2 infected hamsters, mock vaccinated or vaccinated with the combined NSP-2, NSP-14, and Nucleoprotein-based mRNA/LNP vaccine at 4x magnification. Fourteen days post-challenge, the lung tissues were collected and fixed, and 5-μm sections were cut from hamsters and stained with hematoxylin and eosin. The lungs of mock-vaccinated hamsters demonstrate numerous bronchi with bronchiolitis. Lungs of hamsters that received a combined T cell antigens mRNA/LNP vaccine demonstrate mostly normal bronchial, bronchiolar, and alveolar architecture. Significantly reduced degree of inflammation (black arrows) in the lung tissues is found in the hamsters immunized with combined T cell antigens (NSP-2 + NSP-14 + Nucleocapsid) in comparison to the Mock-vaccinated hamsters. Severe lung pathology in Mock-vaccinated hamsters is characterized by an increased degree of inflammation (black arrows) and, significantly, a smaller number of lung vacuoles. The image is taken at 4X, and the scale bar is 100mm. The data represent two independent experiments; the graphed values and bars represent the standard deviation (SD) between the two experiments. The Mann-Whitney test (for two groups) or the Kruskal-Wallis test (for more than two groups) was used for statistical analysis. ns *P* > 0.05, * *P* < 0.05, ** *P* < 0.01, *** *P* < 0.001, **** *P* < 0.0001.

Following virus challenge, mock-vaccinated hamsters progressively lost up to 10% of their body weight within the first week after infection, before gradually returning to their original weight by approximately day 12. Hamsters that received the mRNA/LNP vaccine expressing Spike 2P or Spike 6P were protected against weight loss following virus challenge (*P* ≤ 0.001, Fig. 3B). Conserved T cell antigens mRNA/LNP-based vaccines, NSP-2, NSP-14, and Nucleoprotein also prevented weight loss (*P* < 0.05, Fig. 3B) with NSP-2 was the most protective (only 2% body weight loss), followed by Nucleoprotein (4% body weight loss) and NSP-14 (6% body weight loss). Faster recovery was also observed in hamsters vaccinated with the spike and T cell antigens, as measured by reversal of their weight loss as early as 4–5 days after challenge (*Gray arrows*, Fig. 3B), versus 6–9 days for the mock-vaccinated hamsters (*Black arrows*, Fig. 3B) (see also Fig. 4A). Two other conserved T cell antigens (i.e., NSP-3 and ORF-7a/b) showed a trend toward protection against weight loss, but along with the remaining 5 T cell antigens (i.e., NSP-4, NSP-5–10, NSP-12, Membrane, and Envelope) did not produce any significant protection against weight loss (*P* > 0.05, Fig. 3B). Similar protection data as measured by SARS-CoV-2 viral RNA copy number in hamsters on Days 2, 6, 10, and 14 post-challenge, were observed with substantial reductions in virus titer (up to 4 logs) in animals immunized with NSP-2, NSP-14, Nucleocapsid and the Spike vaccines (**Supplementary Fig. S5B)**. NSP-3 and NSP-4 appeared to confer protection early after challenge (days 2 and 6) but not later. No significant protection was seen with any of the other T cell antigens. These protection data correlated with the level of lung pathology, where reduced COVID-19 pathology was observed in hamsters immunized with NSP-2, NSP-14, Nucleocapsid, and the Spike vaccines compared to those vaccinated with ORF7a/b, NSP-3, NSP-4, NSP-5-NSP-10, NSP-12, Envelope, and Membrane (**Supplementary Fig. S6B)**.

### 4. A NSP-2-, NSP-14-, and Nucleoprotein-based mRNA/LNP combination vaccine conferred robust and broad protection against multiple SARS-CoV-2 variants and sub-variants of concern:

We next determined the protective efficacy of a combination mRNA/LNP vaccine consisting of the NSP-2, NSP-14 and Nucleoprotein T cell antigens (Fig. 4A), against VOCs with various characteristics, including an ancestral wild-type Washington variant (WA1/2020), the highly pathogenic Delta variant (B.1.617.2), and the heavily Spike-mutated and highly transmissible Omicron sub-variant (XBB.1.5). Male golden Syrian hamsters were immunized intramuscularly on day 0 and day 21 with the combination vaccine at a 1 μg/dose for each component (3 μg/dose total) (*n* = 6 per group) or mock-immunized (*n* = 6 per group). Three weeks after the second immunization, animals were divided into groups of 5 hamsters each and challenged intranasally, in both nostrils, with 2 × 10^5^ pfu of the wild-type Washington variant (WA1/2020) (*n* = 6 per group), 1 × 10^5^ pfu of Delta variant (B.1.617.2) (*n* = 6 per group) or 2 × 10^5^ pfu of Omicron sub-variant (XBB1.5) (*n* = 6 per group), based on virus titration studies to determine an optimal effective dose in hamsters (data *not shown*).

Vaccination with the NSP-2, NSP-14, and Nucleoprotein-based combination vaccine substantially prevented weight loss (Fig. 4B), reduced virus titer by several logs (Fig. 4C), and significantly reduced lung pathology (Fig. 4D) following challenge with wild-type Washington variant (WA1/2020), Delta variant (B.1.617.2), and Omicron sub-variant (XBB1.5). Of particular interest, 6 out of 6 hamsters that received the combination vaccine and were challenged with the heavily Spike-mutated and transmissible Omicron sub-variant (XBB.1.5) did not lose any weight and had only marginal weight loss after the Washington and Delta virus challenge, versus up to 13% weight loss in the mock-vaccinated animals. In addition, animals vaccinated with the combination vaccine showed a much faster recovery after challenge (0–3 days) compared to 7 days for the mock-vaccinated animals. Fourteen days post-challenge, lung tissues were collected and fixed, and 8-μm sections were cut from hamsters and stained with hematoxylin and eosin. The lungs of hamsters that received the combined NSP-2, NSP-14, and Nucleoprotein-based mRNA/LNP vaccine demonstrated regular bronchial, bronchiolar, and alveolar architecture (Fig. 4D). A reduced degree of inflammation (black arrows) was observed in the vaccinated group of hamsters in comparison to mock-vaccinated hamsters. In contrast, the lungs of mock-immunized hamsters had acute bronchi with bronchiolitis and adjacent marked interstitial pneumonia. Taken together, these results demonstrate that the combination of NSP-2, NSP-14, and nucleoprotein provides rapid, robust, and broad protection against infection and disease caused by multiple SARS-CoV-2 variants and subvariants of concern.

### 5. A Spike, NSP-2, NSP-14, and Nucleoprotein-based combination mRNA/LNP vaccine induced stronger, faster, and broader protection against multiple variants and sub-variants compared to the Spike-alone-based mRNA/LNP vaccine.

**Figure 5:**
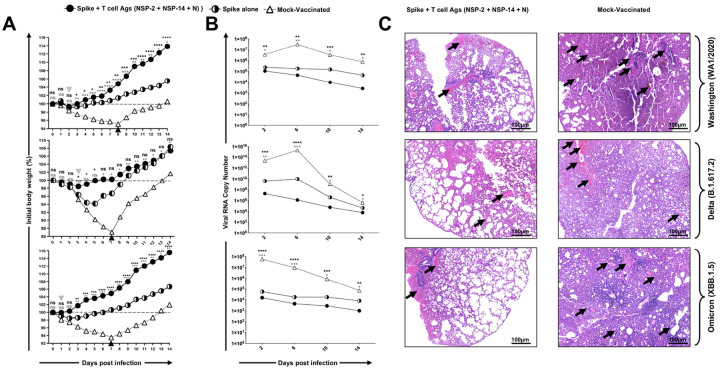
Superior protection induced by combined Spike, NSP-2, NSP-14, and Nucleoprotein-based mRNA/LNP vaccine against the highly pathogenic Delta variant (B.1.617.2): (**A**) Shows percent weight change for 14 days post-challenge normalized to the initial body weight on the day of infection with the highly pathogenic Delta variant (B.1.617.2). The dashed line indicates the 100% starting body weight. (**B**) Virus titers were analyzed at 2-, 6-, 10-, and 14-day post-infection (p.i.) to evaluate vaccine-induced protection against virus replication by comparing viral RNA copies in the hamsters' throats and lungs between the mock and vaccine groups. Viral RNA copies were quantified by RT-PCR and expressed as log_10_ copies per milligram of throat or lung tissue. The graphs show a comparison of viral titers in the lungs of vaccinated and mock-vaccinated hamsters. (**C**) Representative H & E staining images of lung pathology at day 14 p.i. of SARS-CoV-2 infected hamsters, mock vaccinated or vaccinated with the combined Spike, NSP-2, NSP-14, and Nucleoprotein-based mRNA/LNP vaccines at 4x magnifications. The data represent two independent experiments; the graphed values and bars represent the standard deviation (SD) between the two experiments. The Mann-Whitney test (for two groups) or the Kruskal-Wallis test (for more than two groups) was used for statistical analysis. ns *P* > 0.05, * *P* < 0.05, ** *P* < 0.01, *** *P* < 0.001, **** *P* < 0.0001. The upper and lower asterisks refer to comparisons between the combination vaccine versus mock-vaccinated and Spike-alone vaccines, respectively.

We next investigated whether the inclusion of NSP-2, NSP-14, and Nucleoprotein together with Spike would improve the protective efficacy of a Spike-alone vaccine, representing the current standard of care. For this experiment, we chose the prefusion Spike protein stabilized by two prolines (Spike-2P) to match the clinically proven Spike mRNA/LNP-based vaccines. We first demonstrated the functionality of the individual mRNA components, as measured by expression of the four proteins after *in vitro* mRNA transfection of human epithelial HEK293T cells (*white arrows*, **Supplementary Fig. S4**). The co-transfection of the four mRNA vaccines together did not result in any apparent competition, as all four antigens were expressed equally *in vitro* (data *not shown*). The efficacy of the Spike, NSP-2, NSP-14, and Nucleoprotein combination vaccine was compared to the Spike-alone vaccine at a dose of 1 μg/component against multiple variants (Fig. 5A). Hamsters that received the combination vaccine were substantially better protected from weight loss compared to those that received the spike-only vaccine after challenge with the wild-type Washington variant (USA-WA1/2020) (Fig. 5A, Top Panel), highly pathogenic Delta variant (B.1.617.2) (Fig. 5A, Middle Panel), or highly transmissible Omicron sub-variant (XBB.1.5) (Fig. 5A, Bottom Panel). This superior protection was denoted by lower levels of weight loss and faster recovery (0–2 days versus 4–6 days). As expected, the mock-vaccinated hamsters rapidly lost weight and did not regain weight until 7 to 8 days after the challenge. The virus titers determined on days 2, 6, 10, and 14 post-challenge confirmed the significant reduction in viral burden conferred by the combination vaccine versus mock-vaccinated controls (up to 8 logs) and the spike-alone vaccine (up to 2 logs) following challenge (Fig. 5B). Histopathological analysis revealed that the lungs of hamsters receiving the combination vaccine exhibited standard bronchial, bronchiolar, and alveolar architecture (Fig. 5C, *Left Panel*). In contrast, considerable pathological changes, including bronchitis and interstitial pneumonia, are evident in the lungs of mock-immunized hamsters on 14 days post-challenge (Fig. 5C, *Right Panel*). Together, the results (*i*) demonstrate that the combined Spike, NSP-2, NSP-14, and Nucleoprotein-based mRNA/LNP vaccine induced stronger and broader protection against multiple variants and sub-variants versus the spike-only vaccine; and (*ii*) suggest that T cell responses directed toward the conserved T cell antigens may have provided broader protection in the face of immune escape by the heavily Spike-mutated variants, compared to the Spike-alone-based mRNA/LNP vaccine.

### 6. Robust and long-lasting protection against the highly pathogenic SARS-CoV-2 Delta (B.617.2) and recently circulating SARS-CoV-2 Omicron KP.3 sub-variant induced by vaccination:

**Figure 6:**
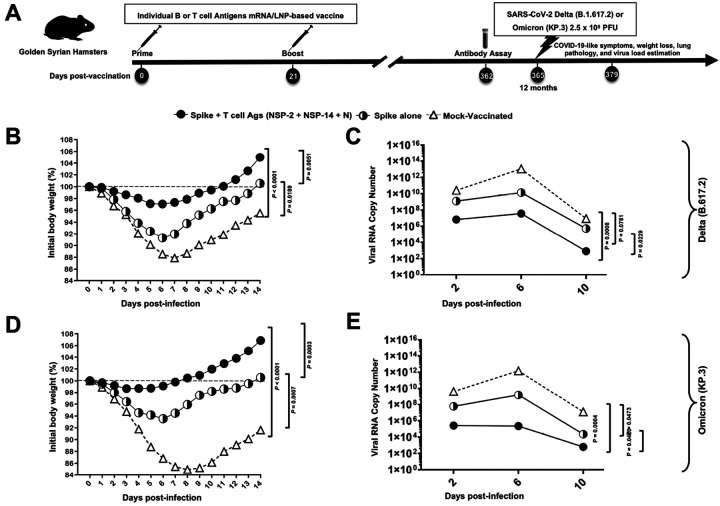
Robust protection induced by combined NSP-2, NSP-14, and Nucleoprotein-based mRNA/LNP vaccine after one year post vaccination against currently circulating SARS-CoV-2 Omicron KP.3 and highly pathogenic SARS-CoV-2 Delta variant in the hamster model: (**A**) Experimental design and timeline to study the vaccine efficacy in golden Syrian hamsters vaccinated with a combination of Spike, NSP-2, NSP-14, and Nucleoprotein-based and Spike alone based mRNA/LNP vaccine. Hamsters were immunized intramuscularly twice on day 0 (prime) and day 21 (boost) with the combined Spike, NSP-2, NSP-14, and Nucleoprotein-based (*n* = 6 per group), Spike-alone (*n* = 6 per group), or mock-vaccinated (*Mock*, *n* = 6 per group). One year (377 days) after booster vaccination, vaccinated and mock-vaccinated hamsters were intranasally challenged (in both nostrils) with 2.5 × 10^^5^ pfu of the current circulating Omicron KP.3 variant or the highly pathogenic Delta variant (B.1.617.2). COVID-19-like symptoms, lung pathology, weight loss, and virus load were assessed for 14 days post-challenge. For the SARS-CoV-2 Delta variant-specific challenge (**B**), the percent weight change over 14 days post-challenge, normalized to the initial body weight on the day of infection, is shown. The dashed line indicates the 100% starting body weight. (**C**) Viral RNA Copy number shown at 2-, 6-, 10-, 14-days post-infection (p.i.) for Delta variant specific challenge. For the SARS-CoV-2 Omicron KP.3 variant-specific challenge, (**D**) percent weight change for 14 days post-challenge, normalized to the initial body weight on the day of infection, is shown. The dashed line indicates the 100% starting body weight. (**E**) Omicron KP.3 variant-specific viral RNA Copy number shown at 2-, 6-, 10-, 14-days post-infection (p.i.). The data represent two independent experiments; the graphed values and bars represent the standard deviation (SD) between the two experiments. The Mann-Whitney test (for two groups) or the Kruskal-Wallis test (for more than two groups) was used for statistical analysis. ns *P* > 0.05, * *P* < 0.05, ** *P* < 0.01, *** *P* < 0.001, **** *P* < 0.0001.

Next, we have evaluated the long-term protection of the combined Spike-, NSP-2-, NSP-14-, and Nucleoprotein-based vaccine versus Spike-alone delivered at a dose of 1 μg of mRNA/component. At one year (i.e., 365 days) following vaccination, separate groups of hamsters were challenged with either the highly pathogenic SARS-CoV-2 Delta (B.1.617.2) or the recently circulating variant SARS-CoV-2 Omicron (KP.3) at 2.5 × 10^5^ pfu (Fig. 6A). Strong protection against both virus strains, as measured by weight loss (Fig. 6B, D) and virus titration (Fig. 6C, E), was induced by the combination vaccine. In contrast, the Spike alone-based mRNA/LNP vaccine induced more modest protection. Consistently robust and superior protection was observed at 3-, 6-, 9-, and 12-month post-vaccination against challenge with SARS-CoV-2 Delta (B.1.617.2) (see **Supplementary Fig. S7** and data not shown).

### 7. Enriched polyfunctional lung-resident antigen-specific CD4^+^ and CD8^+^ T cells and neutralizing antibodies are induced by the combined Spike, NSP-2, NSP-14, and Nucleoprotein-based combination mRNA/LNP vaccine:

**Figure 7:**
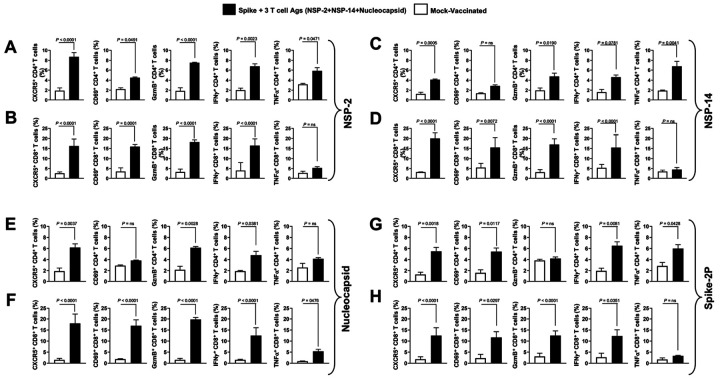
Lung-resident antigen-specific functional CD4^+^ and CD8^+^ T cells induced by the combined NSP-2, NSP-14, and Nucleoprotein-based mRNA/LNP vaccines in the hamsters after one year post vaccination: The panel shows average frequencies of functional CD4^+^ and CD8^+^ T cells after one year post vaccination in the lungs of hamsters vaccinated with the combined NSP-2, NSP-14, Nucleoprotein, and Spike-based mRNA/LNP vaccines. The graphs depict the levels of (**A** & **B**) NSP-2-specific, (**C** & **D**) NSP-14-specific, (**E** & **F**) Nucleoprotein- and (**G** & **H**) Spike-specific CD4^+^ and CD8^+^ cells present in the lungs at 14 days after challenge with the Omicron KP.3 variant in vaccinated (black bars) and mock-vaccinated (open bars) animals. The data represent two independent experiments; the graphed values and bars represent the standard deviation (SD) between the two experiments. Multiple t-tests were used to analyze data. Results were considered statistically significant at *P* < 0.05. The Mann-Whitney test (for two groups) or the Kruskal-Wallis test (for more than two groups) was used for statistical analysis.

Finally, we determined whether the observed rapid and broad protection against SARS-CoV-2 disease and infection in hamsters vaccinated with the combined Spike-, NSP-2-, NSP-14-, and Nucleoprotein-based mRNA/LNP vaccine was associated with antigen-specific lung-resident T cell responses and neutralizing antibodies. Lungs from vaccinated and mock-vaccinated hamsters were collected 2 weeks after SARS-CoV-2 challenge, and cell suspensions were stimulated with pools of 15-mer peptides overlapping NSP-2, NSP-14, Nucleoprotein, or Spike. The frequency and function of antigen-specific CD8^+^ and CD4^+^ T cells were compared in vaccinated protected hamsters versus mock-vaccinated unprotected hamsters (Fig. 7). The data showed that the combination vaccine elicited robust NSP-2- (Fig. 7A, B), NSP-14-(Fig. 7C, D), Nucleoprotein-(Fig. 7E, F), and Spike-specific (Fig. 7G, H), CD4^+^ (Fig. 7A, C, E, G), and CD8^+^ T (Fig. 7B, D, F, H) cell responses, as measured by IFN-g, Granzyme B, CD69, TNFa, and CXCR5 expression, compared to the mock-vaccinated animals.

Since the combined Spike-, NSP-2-, NSP-14-, and Nucleoprotein-based mRNA/LNP vaccine induced strong NSP-2-, NSP-14-, and Nucleoprotein-specific CXCR5^+^CD4^+^ T_FH_ cells, we determined whether the combination vaccine would induce better Spike-specific antibody responses. Serum samples were collected after vaccination and before the viral challenge for analysis by ELISA and neutralization assays. Higher titers of Spike-specific IgG-specific antibodies were detected in 5 out of 5 hamsters that received the combination vaccine compared to hamsters that received the Spike-alone vaccine (*not shown*). Similarly, higher levels of neutralizing antibodies were elicited by the combination vaccine compared to the Spike-alone-based mRNA/LNP vaccine (Table 1). Modest, but consistently higher neutralizing antibodies (up to 4-fold) were observed against the Delta, Washington, and Omicron variants, suggesting that the combination vaccine may have provided additional T cell helper responses, resulting in a more potent antibody response.

## DISCUSSION

The Coronavirus disease 2019 (COVID-19) pandemic has created one of the most significant global health crises in nearly a century^1, 2, 3, 4, 5, 6^. The number of confirmed SARS-CoV-2 cases has reached over 777 million, and COVID-19 has caused almost 7.2 million deaths^1, 5, 6^. As of July 2025, the world is entering its sixth and half year of a persistent COVID-19 pandemic, fueled by the continuous emergence of heavily Spike-mutated and highly contagious SARS-CoV-2 variants and sub-variants that: (*i*) Escape immunity induced by the current clinically proven Spike-alone-based vaccines; (*ii*) Disrupt the efficacy of the COVID-19 booster paradigm^7, 9, 10, 52, 53, 54^; and (*iii*) Outpace the development of variant-adapted Spike-alone vaccines^1, 4, 5, 6, 23^. This bleak outlook of a prolonged COVID-19 pandemic underscores the urgent need for developing a next-generation, broad-spectrum CoV vaccine capable of conferring strong cross-variant and cross-strain protective immunity to prevent immune evasion and breakthrough infections, thereby ending the COVID-19 pandemic^4^.

In the present pre-clinical vaccine study, employing *in silico, in vitro*, and *in vivo* approaches, we screened the SARS-CoV-2 genome for conserved, common viral antigens that could serve as targets for protective T cell-mediated immunity. We identified three such antigens: NSP-2, NSP-14, and Nucleoprotein. Here, we demonstrate that a combination mRNA/LNP vaccine, consisting of Spike, NSP-2, NSP-14, and Nucleoprotein, induces superior, durable, broad, and cross-protective immunity compared to a Spike-only vaccine, representative of the current standard of care, against several highly contagious and heavily Spike-mutated SARS-CoV-2 variants and subvariants. The benefit of including NSP-2, NSP-14, and Nucleoprotein was also demonstrated by using a combination vaccine containing the licensed vaccine Spikevax^™^ as the Spike component (*not shown*). These three T cell antigens are: (*i*) Expressed by the early transcribed virus RTC region; (*ii*) Preferentially targeted by human cross-reactive memory CD4^+^ and CD8^+^ T cells associated with protection of asymptomatic COVID-19 patients (i.e., unvaccinated individuals who never develop any COVID-19 symptoms despite being infected with SARS-CoV-2); and (*iii*) selectively targeted by lung-resident enriched memory CD4^+^ and CD8^+^ T cells from SARS-CoV-2 exposed seronegative individuals who were able to rapidly abort the virus replication (i.e., “SARS-CoV-2 aborters”)^33, 34, 35, 36^. Hamsters that received the combination mRNA/LNP vaccine, displayed lower virus load, improved lung pathology, and protection from weight loss caused by various VOCs including an ancestral wild-type Washington variant (WA1/2020), the highly pathogenic Delta variant (B.1.617.2), the heavily Spike-mutated Omicron sub-variants (B.1.1.529 and XBB1.5), as well as the recently circulating Omicron KP.3 variant. The potent and broad cross-protection induced by the combined mRNA/LNP vaccine was associated with enhanced Spike-specific IgG and neutralizing antibodies, as well as enriched lung-resident NSP-2-, NSP-14-, Nucleoprotein-, and Spike-specific CD4 + and CD8 + T follicular helper (T_FH_) cells, cytotoxic T cells (_CTL_), and effector T cells (T_EFF_). The critical role of CD4 + and CD8 + T cells in protection afforded by the combination vaccine is further supported by data showing that T cell depletion abrogates protection in mice (*not shown*). These preclinical findings are consistent with the above reference data from humans and suggest that an alternative broad-spectrum CoV vaccine may be capable of: (*i*) disrupting the current COVID-19 booster paradigm; (*ii*) outpacing the variant-adapted COVID-19 vaccines; (*iii*) ending the ongoing COVID-19 pandemic, and (iv) preventing future CoV outbreaks.

The efficacy of first-generation Spike-alone-based COVID-19 vaccines is threatened by the emergence of numerous immune-evasive SARS-CoV-2 variants and subvariants that can evade protective neutralizing antibody responses^1, 4, 5, 6, 23^. While the Wuhan strain (Hu1) is the ancestral variant of SARS-CoV-2 that emerged in late 2019 in China, Alpha (B.1.1.7), Beta (B.1.351), and Gamma (B.1.1.28) VOCs subsequently appeared in the United Kingdom, South Africa, and Brazil, respectively, between 2020 and 2021^11^. The most pathogenic Delta variant (B. 1.617.2) was identified in India in mid-2021, where it led to a deadly wave of infections^11^. The heavily Spike-mutated Omicron variants and sub-variants (i.e., B.1.1.529, XBB1.5, BA.2.86, JN.1, KP.3, NB.1.8.1, and LP.8.1) emerged from 2021 to 2025, which are less pathogenic but more immune-evasive.^9, 10, 31^. The waning immunity induced by Spike-alone vaccines, as well as the antigenic drift of SARS-CoV-2 variants, has diminished vaccine efficacy against many recent heavily mutated Spike VOCs^4, 31, 55^. Emerging SARS-CoV-2 variants, particularly the Omicron lineages, which exhibit frequent mutations in the Spike protein, evade immunity induced by vaccination or natural infection^56, 57^. Thus, the first-generation Spike-based COVID-19 vaccines must be regularly updated to better match new VOCs. Yet, despite the recent introduction of updated vaccines, breakthrough infections by the most recent highly contagious, and heavily Spike-mutated Omicron sub-variants, XBB1.5, EG.5, HV.1, BA.2.86, JN.1, KP.3, NB.1.8.1, and LPLP.8.1 are contributing to a prolonged COVID-19 pandemic^9, 10, 53^.

This pre-clinical study comprehensively characterized the safety, immunogenicity, and protective efficacy of SARS-CoV-2-derived T cell antigens using a genome-wide screen and delivered as mRNA/LNP-based vaccine candidates. We identified five highly conserved regions in the SARS-CoV-2 single-stranded RNA genome that encode for three structural (Membrane, Envelope, and Nucleoprotein), 11 non-structural (NSP-2, NSP-3, NSP-4, NSP-5–10, NSP-12, NSP-14), and one accessory protein encoded by the open-reading frame, ORF7a/b^38^. Among these conserved viral proteins, we observed that the early-transcribed non-structural proteins, including NSP-2, NSP-7, NSP-12, NSP-13, and NSP-14, from the RTC region, as well as the structural Nucleoprotein, were selectively targeted by (*i*) peripheral blood cross-reactive memory CD4^+^ and CD8^+^ T cells from asymptomatic COVID-19 patients. This is in agreement with our and others reports that detected high frequencies of cross-reactive functional CD4^+^ and CD8^+^ T cells directed toward specific sets of conserved SARS-CoV-2 non-Spike antigens, including NSP-2, NSP-7, NSP-12, NS-13, NSP-14 and Nucleoprotein in unvaccinated asymptomatic COVID-19 patients^5, 24, 25, 26, 27, 28, 29, 30^; and (*ii*) by lung-resident cross-reactive memory CD4^+^ and CD8^+^ T cells associated with rapid clearance of infection in so-called “SARS-CoV-2 aborters”^33, 34, 35, 36, 37^. The vigorous and enriched cross-reactive RTC-specific CD4^+^ and CD8^+^ T-cells mounted by “SARS-CoV-2 aborters” spontaneously “abort” virus infection so rapidly that they never presented detectable SARS-CoV-2 infection, despite constant exposure to the virus^33, 34, 35, 36^. Similarly, we found the NSP-2, NSP-14, and Nucleoprotein, which are incorporated in our combination mRNA/LNP vaccine, were also targeted by enriched lung-resident antigen-specific T follicular helper (T_FH_) cells, cytotoxic T cells (T_CYT_), and effector T cells (T_EFF_) associated with rapid clearance of the virus from the lungs of protected hamsters^58, 59^. These findings suggest that the early-expressed conserved antigens belonging to the RTC region, which are selectively recognized by CD4^+^ and CD8^+^ T cells from asymptomatic COVID-19 patients and “SARS-CoV-2 aborters,” are ideal targets for inclusion in a next-generation CoV vaccine^33, 34, 35, 36^. It is likely that the rapid induction of local mucosal antigen-specific CD4^+^ and CD8^+^ T cells by early-expressed NSP-2, NSP-14, or Nucleoprotein contributed to the fast control of virus replication and reduced lung pathology in vaccinated hamsters. Furthermore, the Nucleoprotein is the most abundant viral protein and one of the most predominantly targeted antigens by T cells in individuals with less severe COVID-19 disease^39, 40^. Our results also align with a previous report, which demonstrated that Nucleoprotein-specific T cell responses were associated with the control of SARS-CoV-2 in the upper airways and improved lung pathology before seroconversion^60^.

The ten selected protein antigens are highly conserved in all VOCs, including the current highly transmissible and most immune-evasive Omicron sub-variants, KP.3, NB.1.8.1, and LP.8.1, which are currently spreading worldwide. In contrast, the Spike protein is heavily mutated in these variants with an accumulated 346 mutations since the ancestral Wuhan strain. This includes up to 60 new mutations in BA.2.86, JN.1, KP.3, NB.1.8.1, and LP.8.1, which are currently spreading worldwide as subvariants of the original COVID-19 virus. The sequences of the protective T cell antigens NSP-2, NSP-14, and Nucleoprotein remain highly conserved in BA.2.86 and JN.1. Of note, the sequence of NSP-14 is fully conserved (100%) in all variants and sub-variants, including the BA.2.86, JN.1, KP.3, NB.1.8.1, and LP.8.1 supporting the vital function of NSP-14 protein in the SARS-CoV-2 life cycle^61, 62, 63, 64, 65, 66^. NSP-4 (527 aa) is a bifunctional protein; the N-terminal domain has a methyltransferase function required for virus replication^61, 62, 63^, and the C-terminal domain has a proofreading exonuclease function in viral RNA 5′ capping and facilitates viral mRNA stability and translation^62, 64, 65, 66^. NSP-2 (638 aa) is a multi-subunit RNA-dependent RNA polymerase (RdRp) that is involved in replication and RNA synthesis^67 68^. Nucleoprotein (419 aa) plays a vital role in identifying and facilitating virus RNA packaging and in regulating virus replication and transcription^69^. The critical role these viral proteins play in CoV replication and their conservation in SARS-CoV-1, MERS-CoV, and animal SL-CoVs from bats, pangolins, civet cats, and camels make them ideal targets for a next-generation vaccine capable of ending the current COVID-19 pandemic and preventing future CoV outbreaks.

Previous preclinical and clinical studies have supported the hypothesis of targeting T cell antigens to broaden vaccine-induced immunity. These include vaccines based on Nucleoprotein using various delivery technologies, such as recombinant protein^70^, DNA vaccines^71^, mRNA^51, 72^, and viral vectors^73, 74, 75^. In these cases, Nucleoprotein-specific T cell responses and protective immunity have been demonstrated in animal models, as well as immunogenicity in humans. Another approach has been to target specific human T cell epitopes using in silico methods for their identification (Vahed, in Press). One vaccine candidate is based on numerous CD8^+^ T cell epitopes linked in tandem, delivered by a self-replicating mRNA vaccine^3, 76, 77, 78^. Another candidate targets discrete epitopes and segments of T cell antigens, delivered by a conventional base-modified mRNA^79^. Antigen-specific immune responses and protective immunity have been demonstrated in animal models, and both vaccine candidates have been or are currently being evaluated in humans in clinical trials. One potential limitation of epitope-based strategies is the breadth of immunity, in terms of both the number of epitopes presented to the immune system and the degree of coverage across the diversity of human HLA haplotypes. Epitope-specific vaccines are also susceptible to immune evasion, as mutations may create mismatches between the epitopes in the vaccine and the circulating virus strains, thereby rendering the vaccine ineffective. In contrast, inclusion of multiple whole T cell antigens provides substantial redundancy of epitopes and allows determinant selection based on individual HLA types. Using computational and informatics approaches, we identified hundreds of putative human CD4 and CD8 epitopes in the three T cell antigens, which collectively cover more than 99% of the diversity of human HLA types (*not shown*).

Although the present study demonstrated the cross-protective efficacy of a combined mRNA/LNP vaccine against multiple VOCs, specific knowledge gaps remain to be addressed. First, the protective efficacy of the combined vaccine was studied in immunologically naïve hamsters. To better simulate a real-world scenario, we will assess the protective efficacy of the combination mRNA/LNP vaccine in hamsters with pre-existing Spike- or SARS-CoV-2-specific immunity^80^. Second, since NSP-2, NSP-14, and Nucleoprotein contain regions of high homology between SARS-CoV-2 and Common Cold Coronaviruses, the role of cross-reactive T cells induced by the combined mRNA/LNP vaccine should also be investigated in animals that have been previously infected with one of the four major Common Cold Coronaviruses (i.e., α-CCC-229E, α-CCC-NL63, βCCC-HKU1 or βCCC-OC43 strains). Third, since the combined mRNA/LNP vaccine substantially reduced viral load in the upper respiratory tract, it may also reduce transmission and should be evaluated^7^. If so, this would be a highly desirable attribute of a next-generation vaccine capable of blocking the transmission cycle. Finally, this report shows that the combination vaccine elicited lung-resident antigen-specific T_FH_, T_CYT,_ and T_EFF_ cells that may have contributed to eliminating lung-infected epithelial cells and interfered locally with virus replication in the lungs, consistent with reports showing cross-reactive memory CD4^+^ and CD8^+^ T cells alone (without antibodies) may have protected SARS-CoV-2-infected patients with B cell depletion from severe disease^81, 82^, and in non-human primate studies showing that SARS-CoV-2-specific T cells reduced viral loads^83^. However, these may not be the only underlying immune mechanisms responsible for the observed cross-protection. Because immunological reagents and monoclonal antibodies (mAbs) are limited in the hamster model, a better understanding of the B and T cell mechanisms of protection induced by the combined mRNA/LNP vaccine would be enhanced by studying it in mice. Our novel ACE2/HLA triple transgenic mouse model, which expresses the human HLA DR and A*0201 alleles, is ideal for examining the role of T effector cells, dissecting early protein expression, antigen presentation, and stimulation of the innate and inflammatory responses.

In summary, this pre-clinical study in the hamster model presents pathological, virological, and immunological evidence that a Spike-, NSP-2-, NSP-14-, and Nucleoprotein-based combination mRNA/LNP vaccine induced durable, stronger, and broader protection against infection and disease caused by various VOCs compared to the Spike mRNA/LNP vaccine alone, thereby creating a superior vaccine than the current standard of care. The observed protection induced by the combined vaccine was associated with the induction of both Spike-specific neutralizing antibodies and T cell antigen-specific lung-resident CD4 and CD8 T_FH_, T_CYT_, and T_EFF_ cells, which persist for more than one year post-vaccination. These attributes hold promise for a superior next-generation broad-spectrum CoV vaccine and warrant evaluation in human clinical trials.

## METHODS

### Human study population cohort and HLA genotyping:

Between January 2020 and December 2023, over 1,100 unvaccinated patients with mild to severe COVID-19 were enrolled at the University of California, Irvine Medical Center, under an approved Institutional Review Board (IRB) protocol (IRB#2020–5779). Written informed consent was obtained from all patients before their inclusion. A positive RT-PCR test defined SARS-CoV-2 positivity on a respiratory tract sample. The unvaccinated COVID-19 patients were enrolled throughout the pandemic irrespective of SARS-CoV-2 variants of concern they are exposed to: The ancestral Washington variant (USA-WA1/2020), alpha, beta, gamma, the highly pathogenic Delta variant (B.1.617.2), or the omicron subvariants B.1.1.529, BA.2.86, XBB1.5, EG.5, HV.1, JN.1, KP.3, NB.1.8.1, and LP.8.1. Patients were genotyped by PCR for class I HLA-A*02:01 and class II HLA-DRB1*01:01: and ended up with 147 that were HLA-A*02:01^+^ or/and HLA-DRB1*01:01^+^. The 147 patients were from mixed ethnicities (Hispanic (28%), Hispanic Latino (22%), Asian (16%), Caucasian (13%), mixed Afro-American and Hispanic (8%), Afro-American (5%), mixed Afro-American and Caucasian (2%), Native Hawaiian and Other Pacific Islander descent (1%). Six percent of the patients did not disclose their race or ethnicity. The disease severity of the COVID-19 patients included in this study was defined based on an earlier study^41, 84^.

### Peptide synthesis:

Peptide-epitopes from twelve SARS-CoV-2 proteins, including 16 9-mer long CD8^+^ T cell epitopes and 13 15-mer long CD4^+^ T cell epitopes that were selected as described previously^5^. Peptides were synthesized (21^st^ Century Biochemicals, Inc., Marlborough, MA), and the purity of peptides was determined by both reversed-phase high-performance liquid chromatography and mass spectroscopy to be over 95%.

### Human Peripheral Blood Mononuclear Cells and T cell Assays:

Peripheral blood mononuclear cells (PBMCs) from COVID-19 patients were isolated from the blood using Ficoll (GE Healthcare) density gradient media and transferred into 96-well plates at a concentration of 2.5 × 10^6^ viable cells per ml in 200μl (0.5 × 10^6^ cells per well) of RPMI-1640 media (Hyclone) supplemented with 10% (v/v) FBS (HyClone), Sodium Pyruvate (Lonza), L-Glutamine, Nonessential Amino Acids, and antibiotics (Corning). A fraction of the blood was kept separate to perform HLA genotyping on only the individuals who were positive for HLA-A*02:01 and DRB1*01:01. Subsequently, cells were stimulated with 10 μg/ml of each one of the 29 individual T cell peptide-epitopes (16 CD8^+^ T cell peptides and 13 CD4^+^ T cell peptides) and incubated in a humidified chamber with 5% CO_2_ at 37°C. Post-incubation, cells were stained for flow cytometry or transferred to IFN-g ELISpot plates (**Supplemental Fig. S1A)**. The same isolation protocol was followed for HD samples obtained before the COVID-19 pandemic (2018). Ficoll was kept frozen in liquid nitrogen in FBS and DMSO (10%). After thawing, HD PBMCs were stimulated similarly for the IFN-g ELISpot.

### Human ELISpot assay and Flow cytometry:

We assessed CD4^+^ and CD8^+^ T-cell response against conserved SARS-CoV-2-derived class-II restricted epitopes by IFN-g ELISpot in COVID-19 patients representing different disease severity categories (**Supplemental Fig. S1A**). All ELISpot assays were performed as described earlier^41^. Similarly, surface marker detection and flow cytometry analysis were performed on the patients after 72 hours of stimulation with each SARS-CoV-2 class I or class II-restricted peptide^41^. The gating strategy for flow cytometry is detailed in **Supplemental Fig. S1B**.

### Viruses:

SARS-CoV-2 specific to six variants, namely (*i*) SARS-CoV-2-USA/WA/2020 (Batch Number: G2027B); (*ii*) Delta (B.1.617.2) (isolate h-CoV-19/USA/MA29189; Batch number: G87167), (*iii*) Omicron (XBB1.5) (isolate h-CoV-19/USA/FL17829; Batch number: G76172), were procured from Microbiologics (St. Cloud, MN). Omicron KP.3 (Isolate hCoV-19/USA/NJ-GBW-GKISBBBB88291/2024) was procured from the BEI resources. The initial batches of viral stocks were propagated to generate high-titer virus stocks. Vero E6 (ATCC-CRL1586) cells were used for this purpose. Procedures were completed using aseptic technique under BSL-3 containment.

### Data and Code Availability:

Human-specific SARS-CoV-2 complete genome sequences were retrieved from the GISAID database, whereas the SARS-CoV-2 sequences for bats, pangolin, civet cats, and camels were retrieved from the NCBI GenBank. The genome sequence accession numbers retrieved from NCBI GenBank are listed in detail in our earlier publication^5, 84^.

### mRNA synthesis and LNP formulation:

Sequences of Spike and 10 T cell non-Spike antigens were derived from the SARS-CoV-2 Omicron sub-variant BA.2 (NCBI GenBank accession number OM617939) Nucleoside-modified mRNAs expressing SARS-CoV-2 full-length of prefusion-stabilized Spike protein with two or 6 proline mutations (mRNA-S-2P and mRNA-S-6P (Size: 3804 bp, Nucleotide Range: 21504 bp - 25308 bp)) and part or full-length ten highly conserved non-Spike T cell antigens (NSP-2 (Size: 1914 bp, Nucleotide Range: 540 bp - 2454 bp), NSP-3 (Size: 4485 bp, Nucleotide Range: 3804 bp - 8289 bp), NSP-4 (Size: 1500 bp, Nucleotide Range: 8290 bp - 9790 bp), NSP-5–10 (Size: 3378 bp, Nucleotide Range: 9791 bp - 13169 bp), NSP-12 (Size: 2796 bp, Nucleotide Range: 13170 bp - 15966 bp), NSP-14 (Size: 1581 bp, Nucleotide Range: 17766 bp - 19347 bp), ORF7a/b (Size: 492 bp, Nucleotide Range: 27327 bp - 27819 bp), Membrane (Size: 666 bp, Nucleotide Range: 26455 bp - 27121 bp), Envelope (Size: 225 bp, Nucleotide Range: 26177 bp - 26402 bp), and Nucleoprotein (Size: 1248 bp, Nucleotide Range: 28206 bp - 29454 bp) were synthesized by *in vitro* transcription using T7 RNA polymerase (MegaScript, Thermo Fisher Scientific, Waltham, MA) on linearized plasmid templates, as reported^42, 85^. Modified mRNA transcript with complete substitution of Pseudo-U was synthesized by TriLink Biotechnologies using proprietary CleanCap^®^ technology. The synthesized polyadenylated (80A) mRNAs were subjected to DNase and phosphatase treatment, followed by Silica membrane purification. Finally, the synthesized mRNA was packaged as a 1.00 ± 6% mg/mL solution in 1 mM Sodium Citrate, pH 6.4. Purified mRNAs were analyzed by agarose gel electrophoresis and were kept frozen at −20°C. The mRNAs were formulated into LNPs using an ethanolic lipid mixture of ionizable cationic lipid and an aqueous buffer system. Formulated mRNA-LNPs were prepared at varying RNA concentrations (1 μg/μL) and stored at −80°C for animal immunizations.

### Confirmation of protein expression by mRNA.

The expression of the target viral protein by the vaccines was confirmed in HEK293T [American Type Culture Collection (ATCC), CRL-3216] cells before testing in animal experiments, and 10^6^ cells were plated in 500 μl culture medium in a 6-well plate on Day 0. Once the cells reached confluency, *HEK*293T cells in six-well plates were either directly transfected with 2 μg of mRNA-LNP or transfected with LNP alone. A transfection mix for mRNA was prepared, and cells were transfected according to the Lipofectamine MessengerMAX Transfection Reagent-specific protocol (Thermo Fisher Scientific, Catalog # LMRNA001).

### Hamster immunization and SARS-CoV-2 variants challenge:

The mRNA/LNP vaccines were evaluated in the outbred golden Syrian hamster model for protection against three SARS-CoV-2 variants and subvariants (Washington, Delta, and Omicron). The Institutional Animal Care and Use Committee approved animal model usage experiments at the University of California, Irvine (Protocol number AUP-22–086). The recommendations in the Guide for the Care and Use of Laboratory Animals, published by the National Institutes of Health, are used for performing animal experiments. The sample size for each animal study (*n* = 6 per group) was determined by power analysis, demonstrating that five hamsters per group were sufficient to produce significant results with a power of greater than 80%.

For variants and subvariants (Washington, Delta, and Omicron challenge, four groups of 6- to 8-week-old male golden Syrian hamsters (6 per group), strain HsdHan: AURA (Envigo, catalog no. 8901M), were vaccinated intramuscularly with individual or combined mRNA/LNP (1 μg, 5 μg, or 10 μg per dose as indicated in Figures) on day 0 (prime) and day 21 (boost). Hamsters that received phosphate-buffered saline alone were used as mock-immunized controls (*Saline*, *Mock*, *n* = 6). The mRNA/LNP vaccines and saline control were administered in 100 μl per injection. Serum samples were collected from all hamsters before the viral challenge to measure vaccine-induced neutralizing antibodies. Three weeks after booster vaccination (week 6), the hamsters were transferred to the ABSL-3 facility and intranasally challenged with the SARS-CoV-2 Delta variant (1 × 10^5^ plaque-forming units [pfu]) or the Washington or Omicron strain (2 × 10^5^ pfu). At the indicated time points, throat swab samples and equivalent portions of the lung tissues were collected for various analyses of vaccine-induced protection. Hamsters were monitored daily to evaluate vaccine-induced protection from body weight loss. Throughout the experiment when blood and swab samples were collected, the hamsters were anesthetized with inhalation anesthesia using 5% isoflurane mixed with 500 ml/min oxygen in an induction chamber. At the experiment end point, the hamsters were euthanized using isoflurane inhalation followed by cervical dislocation in ABSL3. Isoflurane based euthanization method was adopted as approved by the IACUC as it was not feasible to perform euthanization using CO_2_ in the ABSL3 (UCI IACUC Protocol number AUP-22–086).

### Neutralizing assay:

Serum neutralizing activity was examined, as previously reported^57, 86^. Briefly, the assays were performed using Vero E6 cells (ATCC, CRL-1586). Briefly, serum samples were heat-inactivated and serially diluted three-fold (initial dilution, 1:10), followed by incubation with 100 pfu of either wild-type SARS-CoV-2 (USA-WA1/2020) or the Delta strain for 1 hour at 37°C. The serum-virus mixtures were placed onto a Vero E6 cell monolayer in 96-well plates for incubation at 37 °C for 1 hour. The plates were washed with DMEM, and the monolayer cells were overlaid with 200 μL of minimum essential medium (MEM) containing 1% (w/v) methylcellulose, 2% fetal bovine serum (FBS), and 1% penicillin-streptomycin. Cells were then incubated for 24 hours at 37°C. Vero E6 monolayers were washed with PBS and fixed with 250 μl of pre-chilled 4% formaldehyde for 30 min at room temperature, followed by aspiration removal of the formaldehyde solution and twice with PBS. The cells were permeabilized using 0.3% (wt/vol) hydrogen peroxide in water. The cells were blocked using 5% non-fat dried milk, followed by the addition of 100 μL/well of diluted anti-SARS-CoV-2 antibody (1:1000) to all wells on the microplates for 1–2 hours at room temperature. This was followed by the addition of diluted anti-rabbit IgG conjugate (1:2,000) for 1 hour at room temperature. The plate was washed and developed by adding TrueBlue substrate, and the foci were counted using an ImmunoSpot analyzer. Each serum sample was tested in duplicate.

### RNA extraction and RT-PCR quantification of viral RNA copies:

The oropharyngeal swab sample was analyzed for SARS-CoV-2-specific RNA by quantitative RT-PCR (qRT-PCR). As recommended by the Centers for Disease Control and Prevention (CDC), we used ORF1ab-specific primers (forward: 5’-CCCTGTGGGTTTTACACTTAA-3’ and reverse: 5’-ACGATTGTGCATCAGCTGA-3’) to detect the viral RNA level. PCR reactions (10 μl) contained primers (10 μM), cDNA sample (1.5 μl), SYBR Green reaction mix (5 μl), and molecular-grade water (2.5 μl). PCR cycling conditions were as follows: 95°C for 3 min, 45 cycles of 95°C for 5 s, and 60°C for 30 s. For each RT-PCR, a standard curve was included using an RNA standard (Armored RNA Quant^®^) to quantify the absolute copies of viral RNA in the throat swabs.

### Lung histopathology:

Hamster lungs were preserved in 10% neutral buffered formalin for 48 hours before being transferred to 70% ethanol. The tissue sections were embedded in paraffin blocks and sectioned at a thickness of 8 μm. Slides were deparaffinized and rehydrated before staining for H&E for routine immunopathology.

Statistical analysis was performed using GraphPad Prism 10.0 software (GraphPad Software, La Jolla, CA). Nonparametric tests were used throughout this paper for statistical analysis. Data were expressed as the mean ± SD. Comparison among groups was performed using the Mann-Whitney test (two groups). Two-tailed *P* values were denoted, and *P* values <0.05 were considered significant.

## Supplementary Files

This is a list of supplementary files associated with this preprint. Click to download.

- SupplementaryMaterial.pdf

## Figures and Tables

**Table 1:** Neutralizing antibodies at 3-, 6-, 9-, and 12-months post immunization

SARS-CoV-2 Variant *(Time indicates months post-vaccination)*	Spike	Spike + 3 T cell Ags (NSP-2 + NSP-14 + Nucleocapsid)	EC_50_ Fold Increase	P-values
	EC_50_ values	EC_50_ values		
**SARS-CoV-2 Delta (B.617.2)**				
Delta (B.617.2) - 3 months	5000	10971	2.19	0.043
Delta (B.617.2) - 6 months	846	2788	3.24	0.020
Delta (B.617.2) - 9 months	2025	2881	1.42	0.034
Delta (B.617.2) - 12 months	846.8	1164	1.37	0.025
**SARS-CoV-2 Washington (WA/USA)**				
Washington (WA/USA) - 3 months	8395	24201	2.88	0.022
Washington (WA/USA) - 6 months	1559	3564	2.86	0.045
Washington (WA/USA) - 9 months	959	3389	3.53	0.027
Washington (WA/USA) - 12 months	1873	2315	1.23	0.019
**SARS-CoV-2 Omicron (BA.2)**				
Omicron (BA.2) - 3 months	5522	13259	2.40	0.003
Omicron (BA.2) - 6 months	803	2943	3.66	0.025
Omicron (BA.2) - 9 months	335	728	2.17	0.030
Omicron (BA.2) - 12 months	1485	2071	1.39	0.014

Neutralization assays were performed against the SARS-CoV-2 Washington, Delta, and Omicron variants at 3-, 6-, 9-, and 12-months post-vaccination in animals immunized with Spike alone or the combination vaccine containing Spike and T cell antigens. Shown are EC_50_ values, fold increases compared to Spike alone, and P values, as measured using the Student’s t-test.

## Data Availability

All data are available in the main text or the supplementary materials.
